# A perspective by the German NRZ-Authent on information and knowledge management in governmental authorities

**DOI:** 10.1038/s41538-025-00487-8

**Published:** 2025-07-05

**Authors:** Andreas Hofmann, Jonas Rietsch, Ilka Haase

**Affiliations:** 1 National Reference Centre for Authentic Food, Max Rubner-Institut, Kulmbach, Germany; 2 Faculty of Science, The University of Melbourne, Parkville, VIC Australia

**Keywords:** Agriculture, Science, technology and society

## Abstract

Efforts in the filing and organisation of primary data, despite being a pillar of digital transformation, appear to be less pronounced when it comes to unstructured or text-based data. This situation is contrasted by the demand for knowledge management in organisational units operating at the interface between different stakeholders, including, for instance, governmental authorities. Supporting the specific objectives of our institution, we have developed a central platform that serves as an information management system for food. By providing a flexible infrastructure for the management of text-based information, as well as an interactive and intuitive user interface for application and integration into every-day business, we have implemented a system that facilitates building of a data pool of information relevant for food and feed fraud/authenticity. While transforming the institutional information management within the NRZ-Authent, the platform allows access to the data pool by external partners at national and, potentially, European or international level.

## Introduction

Information is one of the central resources for businesses and public administration alike and its life cycle is characterised by the key stages of production, collection, processing, analysis, interpretation, dissemination, storage and disposal. The handling of the information life cycle within institutional environments differs substantially both in terms of commercial vs. public administration and the nature of information (e.g. scientific numerical data vs. textual data/knowledge). Within the sector of public administration, the need for more advanced information management and governance has gradually received more attention over the recent past (see e.g. refs. ^1,2^) and resulted in digital transformation strategies within various areas, ranging from the adoption of the FAIR data principles^3^ and the requirement for research data management plans to governmental open data.

Food and feed fraud remains a persistent challenge and generally describes the intentional adulteration and mislabelling of food in order to achieve an economic gain; recent estimates suggest costs to the global economy at the order of USD 50 billion per year^4^.

Organisational units at the interface between different stakeholders, monitoring institutions as well as political advisors rely on information in order to fulfil their assigned tasks and, ultimately, enable some form of decision-making that leads to an outcome. By reducing uncertainty in the course of such processes, information is a requirement for ‘informed decision-making’^5^. This paradigm also applies to any actor involved in combatting food fraud, including national/governmental authorities. In Germany, a central role in the fight against food and feed fraud has been assigned to the National Reference Centre for Authentic Food (NRZ-Authent) which operates at the interface between the Federal Ministry of Agriculture, Food and Regional Identity, Federal Offices concerned with food and feed as well as the official food control authorities at state level that carry out executive functions. In addition to the development, evaluation and provision of (analytical) methods for the detection of fraudulent and deceptive practices, a central field of activity assigned to the NRZ-Authent is the provision of specialised knowledge with respect to authenticity and integrity of food^6^. This knowledge is drawn on the one hand from the expertise of NRZ-Authent staff, and, on the other hand, informed by scientific and methodological developments as well as processes within public administration, economy and society. Individual information is thereby obtained from different sources and in widely diverging formats.

Whereas a large body of information is already being aggregated and disseminated by different stakeholders, including the European Commission, national and international governmental institutions, interagency collaborations, food business operators and others, the individual data sources are dispersed, their formats of dissemination are diverse and, as a result, interoperability remains a challenge.

In this perspective, the current situation of food and feed fraud information processing from the point of view of the NRZ-Authent as a national authority is briefly introduced and current developments towards an institutional information management system are described. Despite the specific information contents being focused on food and feed fraud/authenticity, the general framework and challenges of information management within a national authority might be similar in other contexts.

## Institutional information and knowledge management

According to estimates over the past 20 years, it can be assumed that the majority of global information is available in unstructured form (e.g. text)^7^. In particular, the proportion of unstructured information within most businesses and governmental authorities is substantial^8^. For this reason, various applications for automated extraction and analysis of texts have been designed in order to recognise meaningful structures in unstructured or barely structured prose texts. This so-called text mining aims at transformation of information from prose texts into structured formats, which can subsequently be subjected to further analysis with respect to content and meaning. The use of automated text mining accelerates the analysis of larger amounts of data and accumulation of relevant information.

Owing to the increased performance of machine learning approaches and the availability of corresponding language models (unidirectional large language models such as GPT^9^, or the bidirectional BERT^10^) the performance of automated text mining has also substantially improved within the recent past. Here, the meaning of words is represented by so-called embedding vectors, allowing, on the one hand, for analysis and searching of contents in prose texts and on the other hand for generation of new content (such as e.g. translation, summaries, etc).

The necessity to adequately manage and file digital technical or scientific data has been appreciated in many scientific and technical areas and efforts to implement such management have been and are under way, albeit the degree of implementation may differ. From a conceptional perspective, the establishment of such data management is in many cases supported by efforts to implement the so-called FAIR principles^3^. However, despite such efforts with respect to technical and scientific data, in many organisations, the treatment of information related to knowledge, insights and situational connections often lacks any similar type of management^11^. In the context of business management, knowledge comprises factual information, expert insight and individual experiences, resting with the individual owners. In organisations and collaborative environments, knowledge is often manifested in repositories, documents, standard protocols and procedures^12^. In the migration from conventional data storage practiced over the past decades towards data management fit for the agility required for the present-day demands, the data need to be converted so that it can be analysed for particular purposes using algorithms customised for the requirements of a particular organisation^13^. Such data management is all too often not sufficiently addressed, resulting in difficulties to extract useful information comprehensively and systematically. This situation is at odds with the frequent calls by governments^14^ to step up development of applications based on artificial intelligence, as development and training of these builds on the availability of properly organised and accumulated data.

The organising of textual (or general) data is summarised by the term information management and includes the systematic process of collection, organisation with respect to institutional policies, filing and re-distribution of data and knowledge, aiming to ensure that data are securely accessible over longer terms and can be effectively utilised to inform daily tasks and decisions. Conceptually, this approach appreciates ‘data’ as a valuable commodity that is further valorised by staff through appropriate linking, resulting in re-usable ‘information’ and, ultimately, ‘knowledge’. In addition to affording central storage and findability, the registration of as of yet undocumented insights obtained within the life cycle of collected data through curation and linking efforts undertaken by a team also constitutes an appreciation of the intellectual efforts contributed by the involved staff members^15^.

The application of data-based mechanisms in daily business operations, and in particular the filing and organisation of primary data, is a pillar of digital transformation^16^ and allows for efficient operations, more informed decisions and more sustainable processes overall. As mentioned above, the fundamental importance of such handling of primary data has widely been recognised in organisations with respect to numerical data (such as, e.g. analytical databases) but is often not yet applied when dealing with other types of data in institutional environments. Examples of software applications that help to organise text-based information include document management systems, news portals, knowledge organisers, etc. In this context, institutional information management needs to ensure that data, information and knowledge are recorded, stored, processed and disseminated in a way that supports the objectives of a particular project or the entire institution.

Technically, the collection and processing of data from different sources is afforded by so-called data aggregators; however, to meet end user expectations, the functionalities and features of data aggregating software applications go beyond the mere data collection, filing and basic processing but also include records management (e.g. editing functions, permission management, activity protocols), document management (e.g. linking of digital documents with entries, association of metadata with documents, filing systems for large/unknown numbers of documents), data mining (e.g. filtering and export functionalities), as well as features to allow collaborative work.

## The NRZ-Authent and its data management portfolio

Following the Regulation (EU) 2017/625 of the European Parliament and Council^17^, the German Federal Ministry of Agriculture, Food and Regional Identity established the NRZ-Authent to support the national official food control authorities in their efforts to combat food and feed fraud^18^. Located at the Max Rubner-Institut (the German Federal Research Institute for Nutrition and Food), the NRZ-Authent is embedded in the federal research structure overseen by the Ministry. In their 2023 research plan (and backed by the 2025 coalition agreement of the federal governement), the Ministry identified digitalisation as a horizontal theme of missions and strategic research areas; it recognised digital data management as well as data economy as important components that must be integrated into all research endeavours within the Ministry’s area of oversight^19^. Importantly, the transformation of processes using digital technology (digitalisation) needs to be underpinned by digitisation, i.e. efforts to convert analogue or verbal information into digital form as well as conceptional and organisational measures.

A substantial number of specific activities of the NRZ-Authent are concerned with the collection, evaluation and dissemination of information with respect to food and feed fraud/authenticity. Particularly, addressing the objectives of (i) evaluation of methods for the detection of fraudulent and deceptive practices, (ii) the coordination of relevant activities and (iii) the provision of expertise on the authenticity and integrity of food products requires the collection, consideration and synthesis of information from fundamentally different sources with diverse timeliness. The role of the NRZ-Authent at the interface of official food control, ministerial governance and industry necessitates substantial resources with respect to procurement, consolidation and provision of information.

## Sources of information for food fraud

An initial stock-taking of information sources that are of importance to the NRZ-Authent by nature of its role as a national authority identified a number of sources that deliver new information in regular intervals and widely varying different formats. This includes, for example, the news summaries of the German national (‘BeoWarn’) and European Union (‘RASFF’) early warning systems, curated summaries of relevant national authorities (e.g., ‘Notes on Food Fraud’ by the Federal Office of Consumer Protection and Food Safety (BVL), Germany; ‘Seismo Info’ by the Federal Food Safety and Veterinary Office (BLV), Switzerland; ‘Monthly Food Fraud Summary Reports’ by the Joint Research Centre (JRC), EU), as well as industry (e.g., ‘Monthly Trend Risk Report’ by the International Featured Standards) and project groups (e.g., reports by the project group prioritising topics based on the ISAR screening tool^20^). In addition, there is also a vast amount of information received at the NRZ-Authent that originates from network contacts within the national official food control.

At a broader level, there are, of course, many more data sources that can provide regular updates or substantial existing data on food authenticity/food fraud. A non-exhaustive compilation of sources and current web links (where available) to such sources are compiled in Table 1, sectioned into the type of source being either (i) a national European authority, (ii) the European Union, (iii) a national world-wide authority, or (iv) a commercial provider, network or association.

**Table 1:** Sources of information regarding food and feed fraud/authenticity that provide regular updates or substantial existing data

Information source	Provider	Contents	Form of distribution
National authorities in Europe			
BeoWarn	BVL, DE	Curated information from ~200 sources since 2017	Monthly newsletter in PDF format, served through a restricted file share system
Notizen zu Lebensmittelbetrug	BVL, DE	Curated information since 2020	Annual newsletter in PDF sent by email
ISAR project group	Members of the project group researching prioritised topics based on evaluation of ISAR (Import Screening for the Anticipation of Food Risks) data; official food control, DE	Background information research for prioritised food topics	Presentation in MS PowerPoint format for members of the project group
Seismo Info	BLV, CH	Curated information from diverse sources	Monthly report in PDF format, served on the publicly available BLV website
European Union			
EU Rapid Alert System for Food and Feed	European Commission, EU	Early warning system for food and feed with information by food control authorities of the member states	(1) Web-based user interface without API but export options for (a) public (RASFF Window) and (b) non-public (iRASFF) Information. (2) Weekly iRASFF annotated reports by the Commission in PDF format (public and non-public information) distributed via the national contact points (3) Monthly reports by the Commission in XSLX format (public and non-public information) distributed via the national contact points (4) Monthly reports by the Commission in PDF format (public and non-public information)
Europol operation OPSON	Europol, EU	Information from joint actions to fight food fraud as well as food stuffs and drinks of inferior quality	Press releases, scientific publications
Monthly Food Fraud Summary Reports	Knowledge Centre for Food Fraud and Quality (KC-FFQ), European Commission, EU	Media reports from sources covered by MedISys, filtered using keywords for food adulteration	Monthly reports in PDF format available from the public website
MEDISYS for food fraud	Wageningen University & Research, NL	Food fraud information extracted from the Europe Media Monitor (JRC, EU)	Web-based user interface, access account required (website)
National authorities world-wide			
FoodSHIELD	US Government	Food fraud and food defense database	Collaborative platform with access for US regulatory authorities, analysis laboratories, academic participants and military (website)
US recall notices and noncompliance data	FDA, US	Information from press releases and other public announcements regarding recalls of FDA-regulated products	Web-based user interface, no export options
Commercial providers, networks and associations			
Agroknow FOODAKAI	Agroknow, Athen, GR	Combined monitoring, evaluation and prevention of food risks	(a) Fee-based food risk information platform (website) (b) Free Email notification of recalls, import warnings, fraud incidents, food safety, inspections, warning letters and risk prediction (c) Free? App (d) Free reports, registration required (website)
Amfori Country Risk Classification	Amfori, Brussels, BE	Geopolitical country risk classification	Publicly accessible reports (website)
Database of Food Fraud Records^32^	The United States Pharmacopeial Convention, Hayward CA, US	Information extracted from scientific literature, media publications, reports of supervisory authorities, court files, reports of professional associations as well as other public sources starting in 1980, ~15.5k entries	Relational database, access modalities unknown. Cf. FoodChain ID Food Fraud Database.
fera HorizonScan	Fera Science Ltd., Sand Hutton, York, UK	Potential and developing problems regarding food safety	Subscription-based database (website)
Food Adulteration Incidents Registry	iDecisionSciences, Washington, D.C., US	Collection of fraud incidents (food and ingredients) extracted from diverse sources, including LexisNexis, PubMed, media reports, FDA recalls, reports of US States, the EU RASFF portal and the START Global Terrorism Database.	Web-based user interface
FoodChain ID (ehem. Decernis) Food Fraud Database	FoodChain ID, Washington, D.C., US	Curated, searchable database with records on fraud incidents to support vulnerability analysis required by GFSI	Subscription-based database (website). Cf. Database of Food Fraud Records.
Food Fraud Risk Information	‘experts for food fraud prevention’ (Admin: karenconstable1: Food Fraud Advisors, Gordon, New South Wales, AU)	Collection of information on fraud incidents and fraud risks sorted by food matrix	Publicly accessible Kanban Board on the cloud service Trello
SGS DIGICOMPLY	SGS Digicomply, Chiassc, CH	Data regarding food safety and food ingredients, regulatory data	Collaborative platform providing information, horizon scanning, compliance evaluation and consultancy (website)
Transparency International Corruptions Perception Index	Transparency International e.V., Berlin, DE	Geopolitical country risk classification	Publicly accessible ranking (website)

A central resource in Europe with respect to regular and automated collection of information is the European Media Monitor^21^ that has been developed and is maintained by the JRC of the European Commission, supporting their research activities in particular with respect to language processing, text classification and analysis. According to information by the JRC, that system monitors 17,000 web sites on a regular basis and processes 300,000 news items in up to 70 languages per day. Building on the European Media Monitor, the JRC offers the publicly available web service EMM-NewsBrief^22^ as well as the platform MedISys^23^ that provides medical information. Both systems offer users the functionality of data aggregation, i.e., based on regular/continuous data entry by the platform’s provider, users can access information through a rich site summary-feed/news channel or browse and search/filter the information using the web interface. Despite MedISys originally intended for providing medical information, the European Food Safety Authority tested the use of this system for automated monitoring of food and feed safety by extending the list of covered sources as well as adding filters for common food and feed hazards^24^. For the time dating back to 2016, the ‘Monthly Food Fraud Summary Reports’ have been collated by JRC Unit ‘Food Integrity’ and are published on a publicly accessible website (see Table 1).

More recent applications by academic groups aim beyond the mere data aggregation and have focused on the sharing of data for prediction of potential food fraud by using approaches such as federated learning^25^ or fraud risk analysis of novel foods^26^, adding to and improving on a number of existing approaches for risk and vulnerability assessment with varying degrees of in-built automation (see Table 2).

**Table 2:** Applications for vulnerability analysis in food supply chains

Application	Provider	Description	Further information
EMAlert^TM^	Battelle Memorial Institute, Columbus, OH, US	Web-based application for food producing industry, license required	Batelle website
Identification and Prevention of Adulteration Guidance Document	American Spice Trade Association, Washington, DC; US	Freely available guide	Document, ASTA website
SSAFE	SSAFE (public-private partnership)	MS Excel spreadsheets, registration required	Website
USP Food Fraud Vulnerability Assessment and Mitigation Plan Guidance Document	U.S. Pharmacopeial Convention, Rockville, MD, US (non-profit organisation)	Freely available guide for identification of vulnerabilities and control plan for risk minimisation	Document
Vulnerability Assessment Tools	Food Fraud Advisors, Gordon, New South Wales, AU	MS Excel spreadsheets, license required	Food Fraud Advisors website
GFSI-recognised certification programme owner guidance documents	Global Food Safety Initiative, Levallois-Perret, FR	Certification programme with generation of standardised documentation	Website
PremiumLab guide to preventing fraud in the food industry	Premiumlab, S.L., Barcelona, ES	Freely available guide	Document

## Requirement for institutional information and knowledge management systems

From an institutional point of view of an agency such as the NRZ-Authent, existing data aggregation systems like those summarised in Table 1 are highly valuable sources of information but, given the individual focus of each of these diverse applications, none of these constitutes a one-stop solution that could be employed to fulfil the agency’s mission. The shortfall is mainly due to the limited coverage inherent in such approaches since, e.g., news disseminated within channels of national governments are not included as sources in applications developed by third-parties.

As part of its mission, the NRZ-Authent needs to broadly gather information in a non-targeted manner since it needs to stay at the forefront of current knowledge to be able to advise the Federal Ministry or support the food control authorities within particular tasks. Also, for imminent questions or problems, information needs to be collated for specific topics.

Supporting the institutional goals such as those outlined above and at the same time driving forward the process of digital transformation, new resources are being developed at the NRZ-Authent and combined into a central platform that serves as an information management system for food authenticity. This system specifically addresses three objectives: (i) creation of a flexible infrastructure for the management of text-based information, (ii) building a data pool of information relevant for food and feed fraud/authenticity by tapping into relevant sources, (iii) provision of an interactive and intuitive user interface for application and integration into every-day business. The resultant platform will transform the institutional information management within the NRZ-Authent and also allow access to the data pool by external partners at national and, potentially, European/international level.

## State of the art and related works

Given that none of the food fraud data aggregation services identified so far (see Table 1) provides or allows for incorporation of all information sources actively used by the NRZ-Authent, and in order to drive forward the digital transformation process as prioritised by the Federal Ministry, we decided to develop a custom-built platform named Food Authenticity Knowledge Tool (FAKT). With respect to the desired functionality, rather than employing a top-down design which might be out-lived very quickly, we decided to evolve the information management system starting from a few central functions and defining a logical framework within which new functionalities can be developed. The most important criteria of the framework include (i) the ability to connect to any sources or external services in the future as the platform evolves and opportunities to link to other services arise; (ii) the ability to re-adjust existing contents if required in order to enable addition of (deep learning-based) analysis features, (iii) the ability to store additional unstructured metadata that may be accessed through the user interface or subjected to particular analysis modules.

The central component of the design is a so-called news item that comprises a title, the information content in prose form and, optionally, a set of metadata. A news item can be linked with a document in PDF format uploaded to the platform or an external source by a uniform resource locator (URL) or digital object identifier (DOI). The management of uploaded documents includes a dynamic storage folder organisation as well as checking for known viruses/malware and file name management upon upload. As a general rule, main content of a news item that is available on external services is not hosted on FAKT; on the one hand, this serves to minimise the required storage capacity, and on the other hand helps to avoid any potential copyright issues, e.g., in the case of reprints of scientific literature.

A permission model based on user/news groups that can be defined dynamically allows for restricted access to groups of news items that have been affiliated with a particular group. Any registered user can enter news items into the system, but certain features relating to maintenance and organisation require the status of a ‘curator’. These role-based permissions are defined by the roles assigned to individual user accounts and the processing of curation requests is implemented by means of a ticket system through which enquiries and post-submission or maintenance requests are managed and executed.

For reasons of content consistency and to support development and application of automated functions, it is an advantage to fix the language of the news item contents. In order to be able to offer access to the information management system to non-German/European partners, the content language has been fixed to English. For manual content entry, the system offers translation of German (and other non-English) language texts into English, supported by a connected large language model (LLM).

Besides on-demand translation, the LLM is used to offer additional features like summarisation to make manual data entry easy and fast. The translation feature is also part of automatic processing pipelines, for example when parsing non-English data from reports. To ensure that mistakes potentially introduced by these automated processing steps can be uncovered and fixed, the original text, as extracted from the data sources, is stored as metadata. The availability of these metadata allows for rerunning analyses with more potent LLMs as they become available. Another application of LLMs in FAKT is the automated extraction of key information, like relevant entities, from news items, which can then be used to offer features like a more targeted semantic search process based on the extracted information. On the one hand, this makes the LLM a central component of the system; on the other hand, it is integrated in a very modular way to allow for an easy transition to other LLMs, which could, for example, be hosted on another server or provided by third parties and accessed via an application programming interface (API).

Whereas all personal data relating to access accounts and authentication are stored in encrypted form, the information content in the database or uploaded documents are not encrypted in order to enable standard database search and filtering methods without attenuation of performance due to decryption steps.

**Fig. 1:**
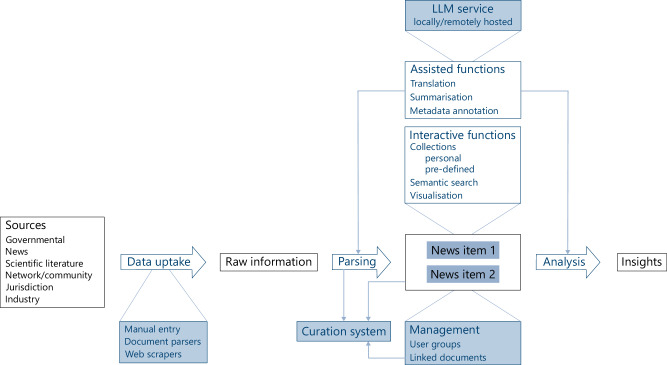
Schematic portfolio of the FAKT information management system.

A schematic summary of the current and planned portfolio of FAKT is given in Fig. 1.

## Current implementation

As a proof-of-principle, several individual functional components of an information management system addressing the above-mentioned concept have been developed and integrated into the web-based application FAKT hosted at our institution. The service provides end users with easy access to the accumulated information from a wide variety of different sources (one-stop solution) and, owing to its extensive data pool, offers the opportunity to test and develop various functions for information analysis, linking, visualisation and interaction.

At present, FAKT utilises four different routes for information uptake: (i) parsing of information from structured reports in PDF formats; (ii) scraping of information from specific web sites; (iii) fetching of information through APIs of dedicated external services; and (iv) by manual entry of news items through the user interface. So far, parsers for three different reports, scrapers for information on two external web sites and modules querying APIs of another three external web sites have been implemented. Through these mechanisms and including the manual entry of information through the user interface, some 30,000 news items have been aggregated over the past year.

The inbuilt curation system offers a quality control mechanism for the knowledge base. It allows any user to suggest changes or hint at low quality/wrong information, which can then be checked by a curator who renders a decision on either modifying the contents of a news item or removing it. Furthermore, a graphical flag is used throughout the service to notify users of automatically extracted or modified content; double-checking of such contents by reading the original contents provided by the source is facilitated in a convenient way through the associated hyperlinks or documents.

With respect to the permission management for content from different sources, two different mechanisms were tested: (i) personal permissions within a model with the discrete levels ‘open’ and ‘confidential’, and (ii) assignment of users to user groups. In our experience, the latter system of user groups proved to be efficient and more suitable as it offers more flexibility and allows for more fine-grained settings, in particular for access to information from sources that require access restrictions to very specific audiences.

**Fig. 2:**
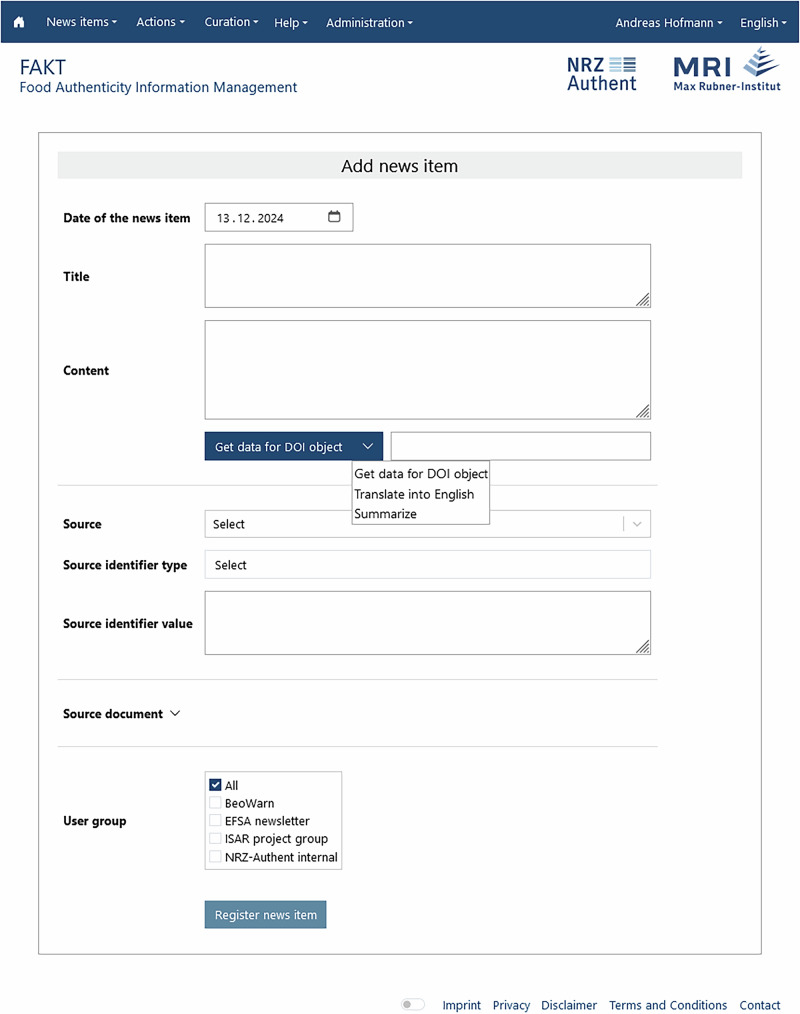
Screenshot of the user interface for data entry.

At present, all users can enter information manually through the user interface (UI) and, once submitted, that content is added directly into the information pool. In contrast, information uptake by means of document parsing, web scraping or querying external databases can only be actioned by users with curator roles. For the manual data entry (see Fig. 2), a number of convenience features have been implemented, including entry of contents just by providing a DOI. Through queries of several external databases, the retrieval of relevant information about that object is attempted and the required fields (and metadata) are prefilled, if successful. Editing of existing contents can be requested by any user who has access to that information based on their user group affiliations. The edit request is logged in an internal ticketing system that is attended to by users with curator roles; the curator then decides whether to allow, modify or deny the suggested edit.

The information from individual or collections of news items can be exported by means of a bibliography data file in RIS-format^27^, enabling convenient integration of data in citation management applications.

## Semantic interoperability

Given the wide variety of sources providing information on food fraud/food authenticity (for examples see Table 1), associated metadata – directly provided or implied – also differ widely. FAKT aims to strike a balance between interoperability on the one hand and information provision/retention on the other by requiring only a restricted set of mandatory metadata but allowing to store any kind of textual metadata associated with a news item. This approach aligns with the bottom-up strategy chosen for the development of FAKT, as it allows addition of new metadata without being constrained by a pre-defined limited metadata scheme.

Mandatory metadata include the source of a news item, a source identifier (e.g., URL, DOI, date, reference number) and the publication date of the news item. Additionally, sources are categorised into source types (e.g. scientific publication, authority, newspaper). The mandatory metadata are used to offer filtering and sorting options that can also be used in conjunction with a search query.

The additional, non-mandatory metadata are stored as a JSON object and may contain arbitrary key-value pairs, although for larger subsets of the data pool (like scientific publications) some fields (such as publication authors) will often be present and, therefore, common keys (‘authors’) are used in such cases.

## Current interactive and collaborative features

Users can leave comments on individual news items that are rendered as blogs, enabling a secure multi-party conversation within the information management application.

News items can be grouped into self-curated collections, thus allowing for generation of data pools that are specific to a given topic. Backed by an embedding model, the items within a collection are used as a starting point for a recommender mechanism that suggests similar news items from the global pool and allows for quick addition of further items to the collection. Personal collections can be shared with other users to allow for collaborative work.

Collections for predefined topics of high interest to a broad audience can be set up by users with curator roles and made available to all users of the system. This feature allows hosting of data pools on topics such as for example the most frequently adulterated foods which can be used as the starting point for research/analysis of particular foods.

Other interactive features include dashboards (e.g., for the RASFF Window notifications) that allow to access and survey data conveniently aiding in the discovery of trends and gaining new insights.

## Practical implementation: transformation of/integration into the everyday work routine

The introduction of a new technology into the existing work routine is generally met with varying levels of receptiveness by employees. A series of measures to achieve the highest possible level of receptiveness, which are also used in various approaches of modern change management, have been important components during development and piloting of this information management system within our institution. These measures include (i) highly intuitive and clearly structured user interfaces that provide relevant features and maximum ease of use; (ii) involvement of users through needs assessment and feedback; (iii) test phases including interested users; (iv) communication and transparency regarding the use and benefits as well as the necessity of the application; (v) introduction, training, and further education for the application; (vi) active usage of the application by management (leading by example).

Integration of an information management system of this nature manifests itself by two different means: On the one hand, the central repository should become the go-to place when searching for particular information or researching relevant topics. On the other hand, valuable information received by the institution or obtained by and located with individual staff should be entered into the system.

In particular, the latter mode of application of such a system is prone to fall subject to hyperbolic discounting^28^, i.e., employees overrate immediate versus future rewards and thus show a reluctance to partake in the shared build-up of the common resource. This behaviour might be further fuelled by employees’ perceived (in-)equity^15^ as the efforts required to integrate such resources into their workflows are experienced as ‘extra’ work that is not compensated for by additional pay or a promotion.

Therefore, in order to foster the build-up of a central resource of this kind and ensure the continued addition of current information, the appointment of a dedicated staff with adequate duties might address challenges of this kind.

## Applicability of FAKT for horizon scanning

In order to scan for dynamically evolving changes in an area of interest, such as in horizon scanning, both unfocussed searches in many different sources and specific research on a particular topic are conducted^29^. From a practical perspective, the unfocussed searches are typically beyond the capabilities of a single person/small team and should build on a large group of individuals^30^. The conceptual design and implementation of FAKT considers these theoretical paradigms and therefore provides a computational tool that can assist in relevant sections of early warning processes and horizon scanning. In fact, the proposed framework for the New Zealand Emerging Risk identification System (ERIS) suggests a roadmap that includes computational tools with the conceptual design as realised in FAKT^31^.

## Strengths and limitations of the study

The information management system has been designed following a ‘bottom-up’ approach that provides maximum flexibility for incorporating new features. Over the first year of operation in an institutional setting, more than 30,000 news items have been aggregated with an estimated 46 h of staff time, owing to the advanced functionalities supporting data entry, data curation and metadata management.

Conceptually, the underlying information management system and many of its features are generally applicable and not thematically bound, i.e., it could in principle be used by any other organisation with a different thematic focus. However, to achieve a maximum of convenience and time efficiency, the implementation of several dedicated features that allow direct incorporation of specific information from sources of interest requires custom-built ‘adaptors’ (parsers, scrapers, etc) and interactive components (e.g. visualisation of data from specific reports).

The results and observations obtained so far obviously refer to the limited period (1 year) of establishing the information management system within our institution. The implementation also involved several rounds of information, training and consultation meetings with all staff in our institution to maximise acceptance and supporting the transformation of work habits. A technical limitation of the present implementation is the lack of more powerful LLMs owing to the currently available hardware infrastructure.

## Conclusion

The information management system described here has been designed considering the mission and type of work of a governmental authority that provides scientific advice to the ministry, acts as a nexus to stakeholders by coordinating and supporting the executing food control authorities as well as coordinating projects that address food and feed fraud issues and involve a diverse range of stakeholders. Considering the diverse range and large amounts of information on food fraud-related topics accumulating at such an authority, a digital management system for non-administrative data was required that can serve as an institutional information management system but quickly be opened to different groups of external participants.

## Future developments

With expansion of the user base both internally and externally, in particular by inviting national food control authorities to subscribe, additional features and more sources will be added to the FAKT information management system. The features planned for addition in the near future include retrieval augmented generation to simplify search queries, development of various visualisation views to improve user-specific data appraisal and email notification to support a ‘news feed’ function available for users preferring to receive digests. The technology for data entry will be further advanced and use vision language models to make incorporation of new sources and news items more generic and convenient. Overall, the existing functions will gradually be upgraded to increase the degree of autonomous processing.

## Data Availability

Access to the web service introduced in this paper can be granted to governmental food control authorities.
